# Epidemiology of acute ice-hockey-related orthopedic fractures in high school- and college-aged players in the United States from 2006–2023

**DOI:** 10.1007/s00068-026-03163-y

**Published:** 2026-04-02

**Authors:** Timothy Reiad, Peter Dinh, Haneef Khan, David Bruni, Elias Chang, Brett Owens, Stephen Marcaccio

**Affiliations:** 1 Department of Orthopaedics, The Warren Alpert Medical School of Brown University, 2 Dudley St, Providence, RI 02905 USA; 2 University of South Carolina School of Medicine, 6311 Garners Ferry Rd, Columbia, SC 29209 USA; 3 University of Michigan, Ann Arbor, MI USA

**Keywords:** Fractures, Ice hockey, Orthopedics, Injury, Trauma, Epidemiology, Sports medicine

## Abstract

**Background:**

This investigation quantified the incidence and injury patterns of acute orthopedic fractures attributed to ice hockey among high-school and college-aged athletes in the United States.

**Methods:**

We performed a retrospective analysis of the National Electronic Injury Surveillance System (NEISS) database focusing on ice hockey fractures in patients aged 14–23 years from 2006 to 2023. The cohort was stratified into high-school-aged (14–18) and college-aged (19–23) groups. Weighted national estimates were calculated to evaluate anatomical sites, fracture subtypes, and hospitalization rates.

**Results:**

An estimated 24,350 ice hockey-related fractures occurred in the study population. Males accounted for 94.8% of cases. Upper extremity and trunk fractures comprised 81.6% of all fractures, with shoulder (27.0%), wrist (19.3%), and lower arm (11.3%) being the most common sites. In comparison to high-school-aged players, college-aged athletes were significantly more likely to sustain hand fractures (OR 2.82; *p* = 0.001) and lower leg fractures (OR 3.67; *p* = 0.042), while less likely to sustain lower arm fractures (OR 0.31; *p* = 0.039). Hospitalization was required for 4.2% of fractures overall, with the neck (82.6%) and upper leg (76.3%) having the highest admission rates, and several areas like the elbow, hand, finger, and knee having admission rates < 1%. Temporal analysis demonstrated no significant linear trend in annual fracture incidence (*p* = 0.18).

**Conclusion:**

Upper extremity and trunk fractures are the dominant injury patterns in high-school-aged and college-aged ice hockey players, predominantly affecting the upper extremity (shoulder, wrist, and forearm). Significant variations in injury location exist between age groups, with college-aged athletes facing distinct risks for hand and knee trauma. These data support the necessity for age-specific protective equipment standards and rule enforcement to mitigate fracture burden.

## Introduction

Ice hockey is characterized by high-velocity skating, rapid changes in direction, and frequent physical contact, creating an environment with inherent injury risks. Globally, the sport engages over 1.6 million registered athletes, including approximately 560,000 annual registrants with USA Hockey [1, 2]. Recent data indicates that the 15–18 age demographic comprises roughly 78,000 US players, with males accounting for approximately 85% of participants [2].

The sport’s regulatory framework allows for a degree of physicality absent in many other athletic disciplines. While major infractions like boarding or charging result in five-minute penalties, minor violations incur only two minutes. Consequently, collisions account for a substantial portion of morbidity, with direct trauma causing 80% of injuries [3]. The combination of puck velocity, stick handling, and body checking drives these injury rates, with the upper extremities frequently identified as the primary injury site in youth populations [4].

The permissive nature of contact rules, paired with the sport’s intensity, elevates the risk of orthopedic fractures [5]. Prior literature indicates that fractures surpass soft tissue injuries-such as lacerations or dislocations-in frequency during competitive play, with the clavicle and metacarpals being particularly vulnerable [6].

Despite the prevalence of these injuries, there is a paucity of data specifically analyzing the transition from high-school-aged to college-aged play. Existing epidemiological studies often aggregate disparate injury types or utilize broad age ranges that obscure developmental trends [7–9]. Furthermore, previous research has often been limited by small sample sizes or single-institution datasets, limiting generalizability. No longitudinal, nationally representative analysis has isolated orthopedic fracture patterns in the 14–23 age bracket over nearly two decades.

To address this gap, this study utilized the National Electronic Injury Surveillance System (NEISS) to evaluate acute fractures in high-school-aged and college-aged ice hockey players from 2006 to 2023. The specific aims were to: (1) characterize fracture distribution by anatomical site; (2) assess temporal trends; and (3) contrast injury patterns between high-school-aged and college-aged groups.

## Materials and methods

### Data collection

This descriptive epidemiological analysis utilized data from the NEISS database, operated by the US Consumer Product Safety Commission (CPSC). The NEISS functions as a stratified probability sample, aggregating data from approximately 100 emergency departments (EDs) representing diverse geographic locations and facility sizes across the United States [7]. This system allows for the calculation of national weighted estimates based on individual case records. The NEISS is one of the few longitudinal surveillance tools capable of capturing severe sports injuries on a national scale [8–15].

### Study design

We queried the database for all injuries related to ice hockey resulting in a diagnosis of fracture between January 1, 2006, and December 31, 2023. The study population was restricted to patients aged 14 to 23 years, chosen to capture the immediate post-checking introduction period (which often begins at the 12U or 14U levels) and the critical transition from youth leagues to more advanced developmental stages characterized by increased body mass and puck velocity [14]. Extracted variables included date of service, patient demographics (age, sex), body part affected, disposition, and narrative descriptions. Non-orthopedic injuries (e.g., skull, facial, or dental fractures) and internal organ trauma were excluded to isolate the specific orthopedic trauma workload on the appendicular and axial skeleton, as craniofacial and dental injuries represent a distinct clinical entity from orthopedic surgical management. As this study utilized de-identified, publicly available data, it was exempt from Institutional Review Board (IRB) oversight.

### Classification

Anatomical sites were categorized according to the NEISS Coding Manual [11]. To facilitate analysis, specific codes were grouped into broader anatomical regions. Upper body categories included the neck, shoulder (clavicle, scapula, proximal humerus), upper arm, elbow, lower arm (radius/ulna), wrist, hand (metacarpals), and fingers. Trunk injuries were divided into upper (thoracic spine, ribs, sternum) and lower (lumbar spine, pelvis, sacrum). Lower extremity categories included the upper leg (femur), knee, lower leg (tibia/fibula), ankle, foot, and toes.

### Statistical analysis

Data analyses were conducted using Stata Statistical Software 18.0 (College Station, TX). We applied the Survey Estimation Module to incorporate NEISS sampling weights, strata, and clustering variables, ensuring accurate national estimates. Statistical significance was defined as *p* < 0.05. Categorical variables were compared using chi-square analysis, while continuous data were assessed via analysis of variance (ANOVA). Logistic regression was used to calculate odds ratios (OR) with 95% confidence intervals (CI) to directly compare unique fracture site distributions between the two target age tiers (college-aged vs. high-school-aged athletes). Weighted estimates are denoted as ‘N’, while raw case counts are denoted as ‘n’.

## Results

### Demographics & incidence

**Fig. 1:**
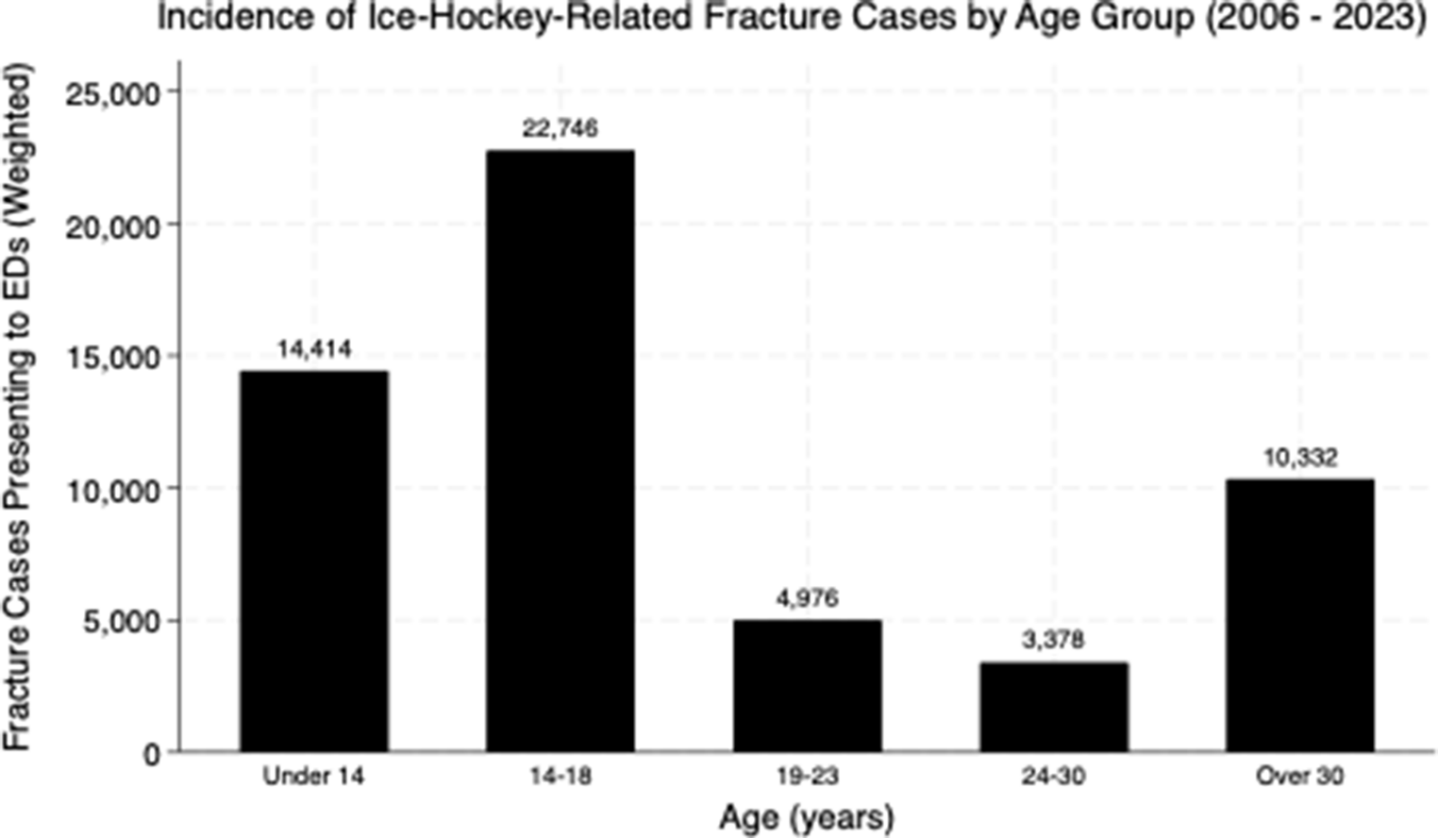
Weighted incidence of ice-hockey-related fractures presenting to US EDs from 2006 to 2023 by age group

**Fig. 2:**
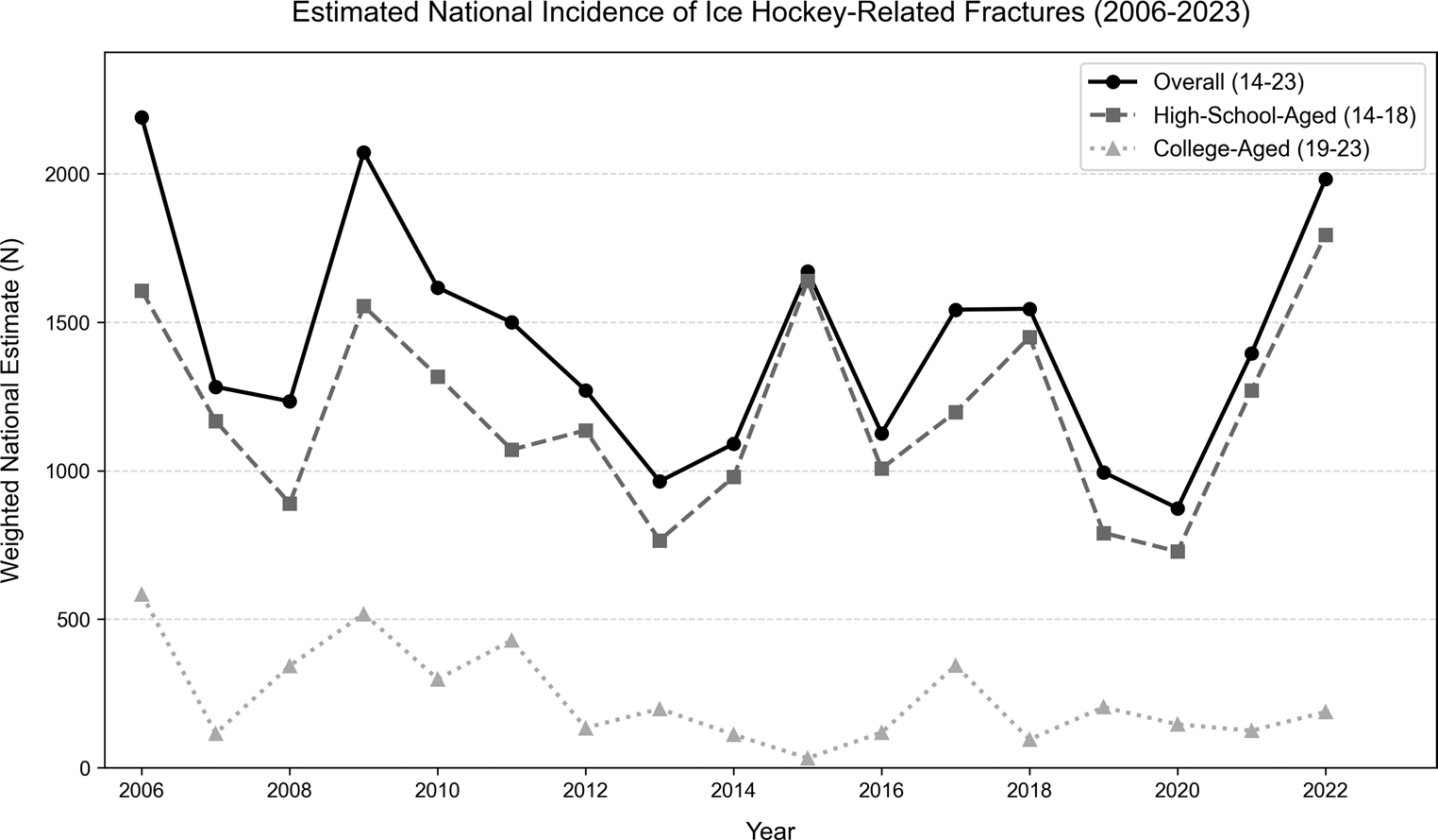
Temporal trends of weighted incidence of ice-hockey-related fractures presenting to US EDs from 2006 to 2023 by age group

The query identified an estimated *N* = 48,448 (*n* = 1,689) total ice hockey-related fractures across all ages during the 18-year period. The specific study cohort of high-school-aged and college-aged individuals (ages 14–23) accounted for 50.3% (*N* = 24,350) of this overall total (Fig. 1). Within this study population, the 14–18 age group represented 42.0% of the overall fracture burden, while the 19–23 age group accounted for 8.2%. Temporal analysis from 2006 to 2023 demonstrated no statistically significant linear trend in annual fracture incidence for this demographic (*p* = 0.18) (Fig. 2).

### Sex distribution

Male athletes in our study cohort sustained the vast majority of fractures (94.8%; *N* = 23,092) compared to females (5.2%; *N* = 1,259) (Table 1). Anatomical distribution varied slightly by sex; males sustained 81.7% of their fractures in the upper extremity/trunk and 18.3% in the lower extremity. Female athletes showed a similar trend, with 79.7% upper extremity/trunk injuries and 20.3% lower extremity injuries.

**Table 1:** Distribution of ice hockey-related fractures by sex, age, and disposition for the study cohort (Ages 14–23)

Variable	Group	Total *N*	Lower Extremity	Upper Extremity and Trunk	*P*-value
Sex	Male	23,092	18.3%	81.7%	0.760
	Female	1,259	20.3%	79.7%	
Age	14–15	10,445	14.4%	85.6%	0.010
	16–17	7,884	15.3%	84.8%	
	18–19	3,039	26.4%	73.6%	
	20–21	1,907	38.8%	61.3%	
	22–23	1,076	21.5%	78.5%	
Disposition	Not Admitted	23,339	17.6%	82.4%	0.018
	Admitted	1,011	35.1%	64.9%	

Summarizes the percentage distribution of ice hockey-related fractures involving the lower extremity versus the upper extremity and trunk across different groups for the localized study cohort

### Anatomic distribution & hospitalization

**Fig. 3:**
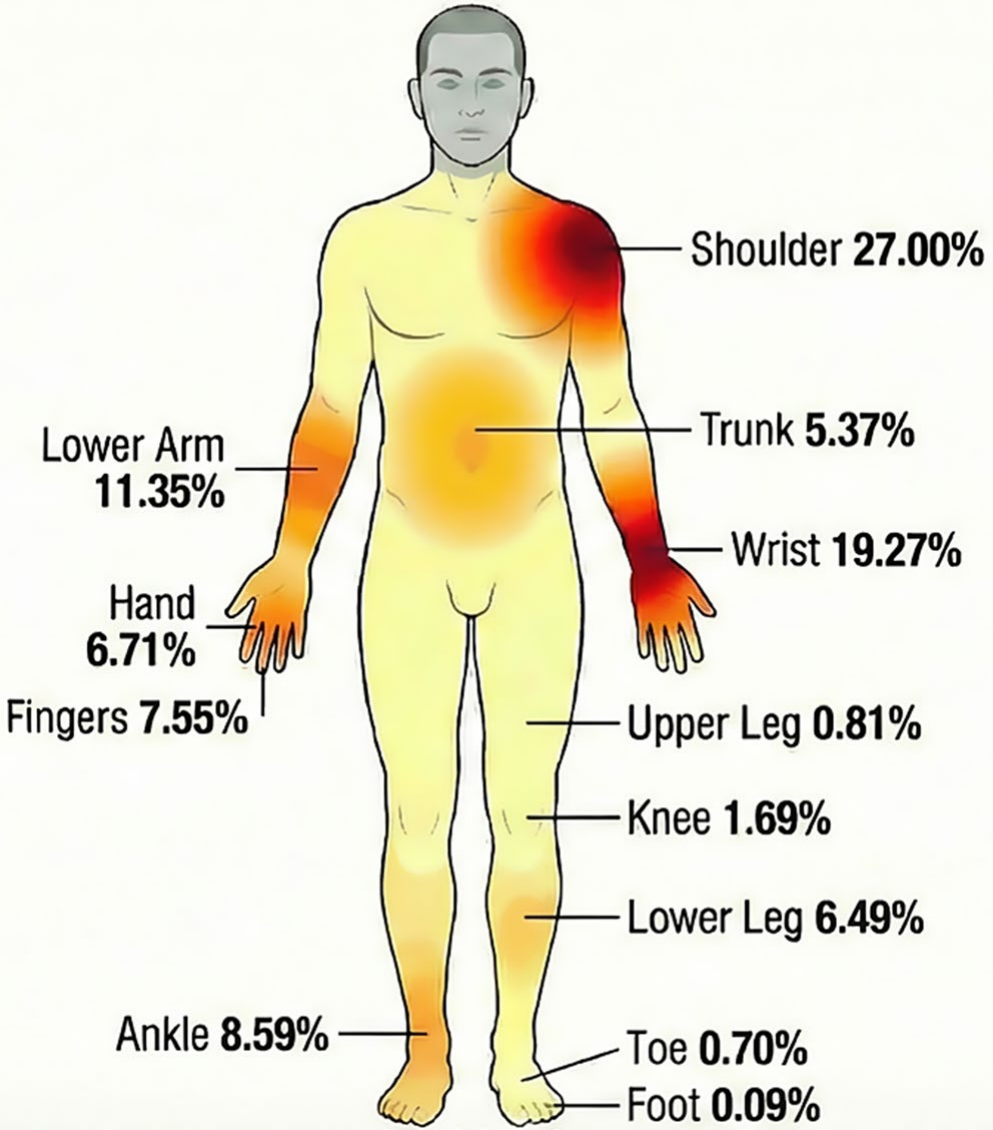
Visual heatmap showing anatomic distribution of weighted high-school-aged and college-aged injuries ice-hockey-related-fractures presenting to US EDs across all from 2006 to 2023

Combined upper body and trunk injuries constituted 81.6% (*N* = 19,875) of the total fracture burden. The shoulder was the most frequent site (27.0%), followed by the wrist (19.3%) and lower arm (11.3%) (Fig. 3; Table 2). Hospital admission was required for 3.3% of upper body/trunk fractures. Admission rates were highest for the neck (82.6%) and lower trunk fractures (19.3%), indicating greater severity in axial skeletal trauma. Lower extremity fractures comprised 18.4% of the total. Within this category, the ankle was the predominant injury site (46.7%), followed by the lower leg (35.3%) and knee (9.2%). Hospitalization was required for 4.2% of fractures overall, with the neck (82.6%) and upper leg (76.3%) having the highest admission rates, and several areas like the elbow, hand, finger, and knee having admission rates < 1% (Table 2) (Table 3).

**Table 2:** Frequency of upper body fracture cases by body part and hospital admission

Region	Body Part	*N* (Fractures)	% of Region	*N* (Admitted)	% Admitted
Upper Body	Lower Arm	2,763	14.9%	174	6.3%
	Upper Arm	325	1.8%	7	2.2%
	Elbow	544	2.9%	0	0.0%
	Finger	1,839	9.9%	55	3.0%
	Hand	1,634	8.8%	0	0.0%
	Neck	195	1.1%	161	82.6%
	Shoulder	6,574	27.0%	40	0.6%
	Wrist	4,693	19.3%	98	2.1%
	**Total Upper Body**	**18**,**567**	**100%**	**535**	**2.9%**
Trunk	Lower Trunk*	607	46.4%	117	19.3%
	Upper Trunk‡	701	53.6%	5	0.7%
	**Total Trunk**	**1**,**308**	**100%**	**122**	**9.3%**
Upper Body + Trunk	**Total Upper Body + Trunk**	**19**,**875**	**81.6%**	**657**	**3.3%**

*Fractures in the lower trunk may involve the lumbar spine, sacrum, coccyx, pelvis, or other nearby bones. ‡ Fractures in the upper trunk may affect the thoracic spine, ribs, or other adjacent bones. Presents the frequency and percentage distribution of upper extremity (UE) and trunk fractures sustained during ice hockey

**Table 3:** Frequency of Lower Extremity Fracture Cases by Body Part and Hospital Admission

Region	Body Part	*N* (Fractures)	% of LE	*N* (Admitted)	% Admitted
Lower Extremity	Ankle	2,092	46.7%	47	2.2%
	Foot	21	0.5%	0	0.0%
	Knee	412	9.2%	0	0.0%
	Leg, lower	1,581	35.3%	157	9.9%
	Leg, upper	198	4.4%	151	76.3%
	Toe	171	3.8%	0	0.0%
	**Total LE**	**4**,**475**	**100%**	**355**	**7.9%**

Reports the frequency and percentage distribution of lower extremity (LE) fractures by body part, along with hospital admission rates for each injury type. Denominator is all LE fractures (4,475)

### Age-specific risk factors

Comparative analysis revealed distinct injury patterns by age (Table 4). College-aged individuals (19–23) exhibited an increased likelihood of hand fractures (OR 2.82; 95% CI: 1.54–5.17; *p* = 0.001) and an increased likelihood of lower leg fractures (OR 3.67; 95% CI: 1.05–12.80; *p* = 0.042) relative to the high-school-aged cohort. Conversely, high-school-aged players were significantly more prone to lower arm fractures (OR 3.22; 95% CI: 1.06–9.77; *p* = 0.039, derived from the inverse of the college OR of 0.31).

**Table 4:** Focused age-specific risk comparisons by key anatomical site (college-Aged vs. high-school-aged)

Anatomical Site	High School (*N*)	College (*N*)	Odds Ratio	*P*-value
Lower Arm	2,590	172	0.31	0.039*
Upper Arm	250	75	1.54	0.655
Elbow	449	94	1.08	0.917
Finger	1,437	402	1.48	0.379
Hand	1,087	547	2.82	0.001*
Neck	161	34	1.09	0.925
Shoulder	5,913	661	0.49	0.064
Wrist	4,188	505	0.56	0.209
Ankle	1,531	561	2.02	0.066
Foot	6	16	14.67	0.064
Knee	323	89	1.41	0.728
Leg, lower	966	615	3.67	0.042*
Leg, upper	198	0	N/A	N/A
Toe	156	15	0.48	0.542

*Indicates statistical significance (*p* < 0.05). Weighted cohort burden is displayed as N. ORs computed based on weighted estimates. Table 4 displays the cross-sectional incidence (weighted N) and direct Odds Ratios specifically comparing College-aged players to High-School-aged players for isolated anatomic disparities

## Discussion

This epidemiological analysis of ice hockey fractures among US high-school-aged and college-aged individuals underscores the significant orthopedic burden predominantly placed on the upper body. Over three-quarters of all fractures occurred in the upper extremity or trunk. While the US age demographics selected correspond roughly to European professional or semi-professional junior club levels, this cohort captures a vital transition window where body size, velocity, and injury potential markedly increase.

### Comparison with prior literature

Our findings substantiate earlier literature regarding age-related shifts in ice hockey injury distributions. National surveillance by Hostetler et al. previously noted that adolescents (ages 12–17) accounted for 47% of all ice hockey injuries [4]. This reflects subsequent findings by Deits et al., who found that over 60% of national emergency department presentations occurred in the 9–14 and 15–18 age groups [12]. In the collegiate athletic demographic, robust surveillance models have historically recognized substantially higher time-loss and game injury rates among collegiate men compared to high school boys [13, 14]. Furthermore, longitudinal studies support our observation that specific upper extremity injury rates increase significantly as youth players transition to mature competitive levels [15].

### Anatomical vulnerability and mechanisms

The shoulder (27.0%), wrist (19.3%), and lower arm (11.3%) represented the overwhelming majority of injuries. This distribution aligns with the biomechanics of the sport. The upper body is frequently subjected to high-impact collisions, such as “boarding” [16, 17]. Furthermore, the instinctive reflex to brace during a fall on a low-friction surface directs significant axial forces directly through the wrist and forearm, propagating to the shoulder girdle. Improving protective gear design to absorb these impacts, such as utilizing wrist guards with non-slip palms [18], and employing specialized falling strategies like increasing negative work during hip flexion to dissipate kinetic energy before impact [19], could meaningfully decrease the incidence of these fractures.

### Age-related variations

A notable finding is the shift in fracture patterns observed in athletes transitioning from high-school-aged to college-aged. College-aged players display lower rates of generalized upper extremity fractures-such as lower arm injuries being significantly less likely (OR = 0.31)-but substantially higher specific loads to the hands and lower legs. This 2.82-fold increase in hand fractures likely reflects a heightened intensity of play involving more severe slash impacts and faster puck velocities at older competitive tiers. The 3.67-fold increase in lower leg fractures is also reflective of the exponentially higher-mass collisions and faster skating speeds seen in more mature players, as body checking and player-to-player contact are associated with substantial increases in lower extremity injury risk [20, 21].

Furthermore, the specific peak in lower extremity injury rates (38.8%) observed in the 20–21 age bracket warrants discussion. This unique anomaly likely reflects a critical inflection point of maximum body mass and peak competitive intensity typically experienced at the junior/sophomore collegiate or semi-professional level, potentially before athletes have fully developed the sophisticated, injury-avoidant skating mechanics refined by older veterans. The subsequent drop in lower extremity injuries among 22–23 year-olds (21.5%) may additionally represent a survivorship bias, where players most prone to high-velocity LE trauma have already dropped out of high-stakes competitive tiers due to prior injuries.

In contrast, high-school-aged players demonstrated a marked 3.22-fold increased risk of lower arm fractures. This may indicate issues with bracing mechanics during falls or checking, as younger athletes may instinctively outstretch their arms defensively rather than absorbing impact with their trunks—a technique often honed at older, elite levels [22, 23]. In addition, differences in glove cuffs and lower arm padding overlap, combined with more frequent slashing infractions in youth leagues not strictly controlled until collegiate or junior play, may leave the radius and ulna disproportionately exposed [24].

### Prevention strategies

The persistence of these injury patterns across an 18-year longitudinal timeline indicates that fracture prevention has seen little statistical evolution. Strict enforcement regarding boarding penalties and a mandate for improved impact-dissipating shoulder pads are pressing needs [24]. Moreover, athletes must be explicitly taught falling mechanics to avoid transferring full loads through locked upper extremities [25].

### Limitations

This study relies on the NEISS database, imposing several constraints. First, its retrospective, ED-based design intrinsically underestimates the true injury burden, excluding fractures managed in private clinics or urgent care settings; furthermore, by focusing exclusively on orthopedic injuries, our findings do not represent the full trauma workload presenting to US EDs. Second, the absence of athlete-exposure data prohibits calculation of precise incidence rates. Third, the abbreviated narrative descriptions preclude granular sub-specification of fracture mechanisms - such as the precise severity of ankle fractures, surgical versus conservative management, or the distinction between puck impact and checking as the causative force. Fourth, neck fractures were retained within the upper extremity category per NEISS anatomical coding conventions, which may marginally affect regional percentage calculations. Finally, NEISS records age but not competitive level; our groupings therefore reflect “high-school-aged” and “college-aged” demographics rather than verified roster status and cannot distinguish injuries sustained in recreational versus elite competitive settings. Additionally, individuals aged 18–19 may fall into either age bracket depending on enrollment status, which cannot be verified from this dataset; these age boundaries were selected to align with standard US educational tiers.

## Conclusion

High-school-aged and college-aged ice hockey players sustain a significant volume of fractures, predominantly affecting the upper extremity (shoulder, wrist, and forearm). The variations between age cohorts — specifically the pronounced propensity for hand and lower leg fractures among college-aged individuals and lower arm fractures in high-school-aged players-suggest that preventative strategies must evolve alongside an athlete’s physical development. Focus on impact-dissipating gear innovations and stricter contact rule enforcement could play a substantial role in reducing this athletic injury burden.

## Data Availability

The datasets analyzed during the current study are publicly available through the U.S. Consumer Product Safety Commission National Electronic Injury Surveillance System (NEISS).

## References

[1] Grill K. The worldwide growth of ice hockey. Coach up nation. 27 July 2016. Accessed November 13, 2024. http://www.coachup.com/nation/articles/ice-hockey-growth.

[2] 2023-24 final registration report. Accessed November 11. 2024. https://cdn1.sportngin.com/attachments/document/2210-1687681/2020-21_Season_USA_Hockey_Final_Registration_Report.pdf.

[3] Daly PJ, Sim FH, Simonet WT. Ice hockey injuries: a review. Sports Med. 1990;10(2):122–31. 10.2165/00007256-199010020-00005. 2204098 10.2165/00007256-199010020-00005

[4] Hostetler SG, Xiang H, Smith GA. Characteristics of ice hockey–related injuries treated in US emergency departments, 2001–2002. Pediatrics. 2004;114(6):e661–6. 10.1542/peds.2004-1565. 15574599 10.1542/peds.2004-1565

[5] A guide to hockey rules & penalties | pure Hockey. Accessed November 27. 2024. https://www.purehockey.com/c/hockey-rules-and-penalties.

[6] Mosenthal W, Kim M, Holzshu R, Hanypsiak B, Athiviraham A. Common ice hockey injuries and treatment: a current concepts review. Curr Sports Med Rep. 2017;16(5):357–62. 10.1249/JSR.0000000000000402. 28902760 10.1249/JSR.0000000000000402

[7] Schroeder T, Ault K, S. Consumer product safety commission. The NEISS sample (design and implementation) 1997 to Present. U; 2001. Available at: https://www.cpsc.gov/s3fs-public/pdfs/blk_media_2001d011-6b6.pdf. Accessed 25 Nov 2024.

[8] Kuczinski A, Newman JM, Piuzzi NS, et al. Trends and epidemiologic factors contributing to soccer-related fractures that presented to emergency departments in the United States. Sports health multidiscip approach. 2019;11(1):27–31. 10.1177/1941738118798629. 10.1177/1941738118798629 PMC6299351 30247999

[9] DeFroda SF, Lemme N, Kleiner J, Gil J, Owens BD. Incidence and mechanism of injury of clavicle fractures in the NEISS database: Athletic and non athletic injuries. J Clin Orthop Trauma. 2019;10(5):954–8. 10.1016/j.jcot.2019.01.019. 31528074 10.1016/j.jcot.2019.01.019 PMC6738494

[10] Caputo P, Mattson DJ. Recreational ice hockey injuries in adult non-checking leagues: A United States perspective. J Sports Sci Med. 2005;4(1):58–65. 24431962 PMC3880085

[11] U.S. Consumer product safety commission (CPSC). National Electronic injury surveillance system (NEISS) coding manual: January 2019. Available at: https://www.cpsc.gov/s3fs-public/2019_NEISS_Coding_Manual.pdf. Published January 2019. Accessed 25 Nov 2024.

[12] Deits J, Yard EE, Collins CL, Fields SK, Comstock RD. Patients with ice hockey injuries presenting to US emergency departments, 1990–2006. J Athl Train. 2010;45(5):467–74. 10.4085/1062-6050-45.5.467. 20831391 10.4085/1062-6050-45.5.467 PMC2938317

[13] Flik K, Lyman S, Marx RG. American collegiate men’s ice hockey: an analysis of injuries. Am J Sports Med. 2005;33(2):183–7. 15701603 10.1177/0363546504267349

[14] Lynall RC, Mihalik JP, Pierpoint LA, et al. The first decade of web-based sports injury surveillance: descriptive epidemiology of injuries in US high school boys’ ice hockey (2008–2009 Through 2013–2014) and national collegiate athletic association men’s and women’s ice hockey (2004–2005 Through 2013–2014). J Athl Train. 2018;53(12):1129–42. 30721630 10.4085/1062-6050-176-17 PMC6365065

[15] Mölsä J, Kujala U, Myllynen P, Torstila I, Airaksinen O. Injuries to the Upper Extremity in Ice Hockey: Analysis of a Series of 760 Injuries. Am J Sports Med. 2003;31(5):751–7. 12975197 10.1177/03635465030310051901

[16] Black AM, Meeuwisse DW, Eliason PH, Hagel BE, Emery CA. Sport participation and injury rates in high school students: A Canadian survey of 2029 adolescents. J Saf Res. 2021;78:314–21. 10.1016/j.jsr.2021.06.008. 10.1016/j.jsr.2021.06.008 34399928

[17] Anderson GR, Melugin HP, Stuart MJ. Epidemiology of Injuries in Ice Hockey. Sports Health. 2019;11(6):514–9. 10.1177/1941738119849105. 31158326 10.1177/1941738119849105 PMC6822215

[18] Jang ES, Park CN, Levine WN, Popkin CA. A Current Concepts Review of Clavicle Injuries in Ice Hockey From Sternoclavicular to Acromioclavicular Joint. Orthop J Sports Med. 2020;8(9):2325967120951413. 10.1177/2325967120951413. 33029542 10.1177/2325967120951413 PMC7520938

[19] Aguiar OMG, Radivojevic O, Potvin BM, Vakili O, Robinovitch SN. Effective stiffness, damping and mass of the body during laboratory simulations of shoulder checks in ice hockey. Sports Biomech. 2024;23(10):1566–77. 10.1080/14763141.2021.1951828. 34319214 10.1080/14763141.2021.1951828

[20] Council on Sports Medicine and Fitness, Brooks A, Loud KJ, et al. Reducing injury risk from body checking in boys’ youth ice hockey. Pediatrics. 2014;133(6):1151–7. 10.1542/peds.2014-0692. 24864185 10.1542/peds.2014-0692

[21] Peterson BJ, Fitzgerald JS, Dietz CC, et al. Division I Hockey Players Generate More Power Than Division III Players During on- and Off-Ice Performance Tests. J Strength Cond Res. 2015;29(5):1191–6. 10.1519/JSC.0000000000000754. 25436625 10.1519/JSC.0000000000000754

[22] Agel J, Dompier TP, Dick R, Marshall SW. Descriptive Epidemiology of Collegiate Men’s Ice Hockey Injuries: National Collegiate Athletic Association Injury Surveillance System, 1988–1989 Through 2003–2004. J Athl Train. 2007;42(2):241–8. 17710172 PMC1941284

[23] Williamson RA, Kolstad AT, Nadeau L, Goulet C, Hagel B, Emery CA. Does Increasing the Severity of Penalties Assessed in Association With the Zero Tolerance for Head Contact Policy Translate to a Reduction in Head Impact Rates in Youth Ice Hockey? Clin J Sport Med Off J Can Acad Sport Med. 2022;32(6):e598–604. 10.1097/JSM.0000000000001063. 10.1097/JSM.0000000000001063 35981453

[24] White CA, O’Connor SJ, Sestak TR, Fox ES, Cagle PJ. Shoulder injuries in ice hockey players: Prevalence, common management, and return to play. J Orthop. 2023;35:145–9. 10.1016/j.jor.2022.11.017. 36483481 10.1016/j.jor.2022.11.017 PMC9723655

[25] Tuominen M, Stuart MJ, Aubry M, Kannus P, Parkkari J. Injuries in men’s international ice hockey: a 7-year study of the International Ice Hockey Federation Adult World Championship Tournaments and Olympic Winter Games. Br J Sports Med. 2015;49(1):30–6. 10.1136/bjsports-2014-093688. 25293341 10.1136/bjsports-2014-093688 PMC4316846

